# An extensive real-world in field tomato image dataset involving maturity classification and recognition of fresh and defect tomatoes

**DOI:** 10.1016/j.dib.2023.109688

**Published:** 2023-10-15

**Authors:** Tania Khatun, Abdur Razzak, Md. Shofiul Islam, Mohammad Shorif Uddin

**Affiliations:** a Department of Computer Science and Engineering, Daffodil International University, Dhaka, Bangladesh; b Department of Computer Science and Engineering, Jahangirnagar University, Dhaka, Bangladesh

**Keywords:** Tomato dataset, Agriculture, Image recognition, Deep learning, and Computer vision

## Abstract

Tomato, a fruiting plant species within the Solanaceae family, is a widely used ingredient in culinary dishes due to its sweet and acidic flavor profile, as well as its rich nutritional content. Recognized for its potential health benefits, including reducing the risk of coronary artery disease and specific types of cancer, tomatoes have become a staple in global cuisine. Traditional methods for tomato maturity assessment, harvesting, quality grading, and packaging are often labor-intensive and economically inefficient. This paper introduces an extensive dataset of high-resolution tomato images collected over an eight-month period from the demonstration fields of Sher-E-Bangla Agricultural University in Dhaka, Bangladesh, in collaboration with plant breeding experts of the same university. The dataset was meticulously curated to ensure precision and consistency, encompassing various stages of tomato maturity, including images of both fresh and defective tomatoes. This dataset is a valuable resource for researchers, stakeholders, and individuals interested in tomato production in Bangladesh, providing a robust foundation for leveraging computer vision and deep learning techniques in the agriculture sector. The dataset's potential applications extend to automating tasks such as robotic harvesting, quality assessment, and packaging systems, ultimately enhancing the efficiency of tomato production processes.

Specifications Table

**Table:** 

Subject	Computer science
Specific subject area	Image detection, Robotic harvesting, Image categorization, Ripeness analysis
Data format	Raw images
Type of data	JPEG
Data collection	In collaboration with an expert in the field from Sher-E-Bangla Agricultural University in Dhaka, Bangladesh, images were captured between September 22 and April 23 from the demonstration grounds of the Horticulture Department at the university. It's worth noting that this dataset is entirely new, and no prior research has been conducted using it.
Data source location	**Location:** Sher-E-Bangla Agricultural University **Zone:** Sher-E-Bangla Nagar, Dhaka-1207 **Country:** Bangladesh
Data accessibility	**Repository name:** Mendeley Data **Data identification number:**10.17632/s42kpg8h37.1 **Direct URL to data:**https://data.mendeley.com/datasets/s42kpg8h37/1 **Instructions for accessing these data:** Adhering to the appropriate citation guidelines is crucial when utilizing these datasets.

## 1 Value of the Data

• Robotic harvesting represents an advanced agricultural technology that offers the potential for substantial enhancements in both quality and productivity, while concurrently reducing production costs and minimizing delays [1]. Achieving optimal results with robotic harvesting hinges on harvesting fruits and vegetables precisely at their peak ripeness; otherwise, substantial losses can be incurred. Timely identification of the appropriate harvest time is thus imperative. In the case of tomatoes, harvest timing is conventionally determined based on their skin color [2]. This dataset encompasses data for tomato maturity identification by categorizing it into two subsets: mature and immature tomatoes, based on their surface color complexion. By analyzing the peel color of these images, machines can effectively distinguish between mature and immature tomatoes in real-world scenarios.
• Due to the lengthy and time-consuming process of transportation tomatoes become defective very easily which people are not willing to buy. For agricultural production, fruit processing, and packing businesses, the detection of defective fruits is extremely important because it can bring significant economic ramifications [3]. In Bangladesh, the detection of fresh and defective fruits is often performed manually, a labor-intensive and inefficient process for farmers. Hence, there is a compelling need to develop a novel classification model capable of autonomously identifying fruit defects without human intervention, reducing costs and production time. This dataset comprises tomato quality grading data that visually represents both fresh and defective tomatoes. Training machines with this dataset empowers them to readily identify and categorize fresh and deteriorating tomatoes at an early stage.
• Beyond its immediate applications, researchers can leverage the assembled dataset for various computer vision, machine learning, and deep learning approaches. These techniques hold promise for addressing multiple facets of tomato production, encompassing harvesting optimization, freshness forecasting, packaging automation, and other automated solutions.

## 2 Data Description

Tomatoes are widely cherished and nutritionally rich crops cultivated across Bangladesh. Traditionally, they have been primarily grown as a winter vegetable in our nation. Nevertheless, the Bangladesh Agricultural Research Institute (BARl) has recently introduced some varieties suitable for summer cultivation.

**Fig. 1:**
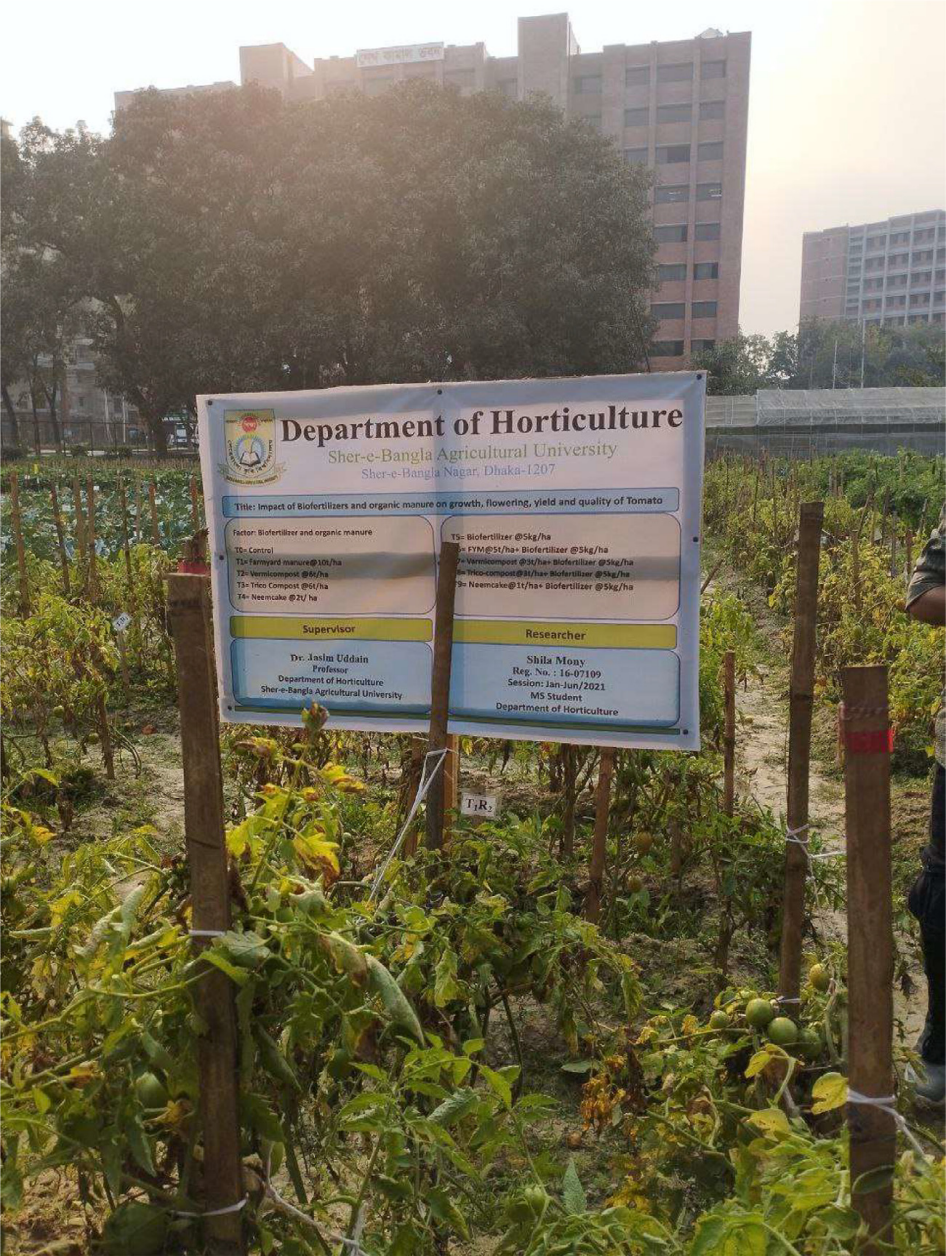
The real tomato field from where data were collected.

The images used in this study were meticulously collected from the Horticulture Department's exhibition grounds at Sher-E-Bangla Agricultural University in Dhaka, Bangladesh, spanning from September 22 to April 23 in collaboration with an expert from the same university. Fig. 1 shows an image of the actual field conditions where our data was gathered. The primary challenge encountered during data collection pertained to capturing images amidst noisy backgrounds and uneven lighting conditions.

Tomato production in Bangladesh is on the rise, offering farmers an additional source of income. Nevertheless, a significant challenge arises from improper storage practices, resulting in substantial losses for farmers. To mitigate these losses, it is crucial to closely monitor the maturity of tomatoes. The condition of vegetable storage and ripening is intricately linked to its level of maturity. In addressing this concern, modern methods have outperformed manual approaches in terms of accuracy, precision, time efficiency, cost-effectiveness, and non-destructiveness.

To facilitate these advancements, in this paper, we have introduced two datasets. The first dataset is called the Tomato Maturity Detection Dataset, and the second is the Tomato Quality Grading Dataset. The level of ripeness and quality are closely associated with the intensity of redness in color and the prominence of flavor [2]. Each of these dataset folders is further split into two subfolders: the original dataset and the augmented dataset. The original dataset folder contains images directly captured with the camera, while the augmented dataset folder contains images generated from the original dataset through data augmentation processes using the software.

Both the original and augmented datasets within the Tomato Maturity Detection Dataset are categorized into two groups: Mature Tomatoes and Immature Tomatoes. Similarly, both the original and augmented datasets within the Tomato Quality Grading Dataset are categorized into two groups: Fresh Tomatoes and Defect Tomatoes. Each of these folders contains relevant tomato images. The images collected come in sizes of 765 × 1024 pixels and 1280 × 957 pixels. [4], Table 1 provides a description of mature and immature tomato categories within the dataset.

**Table 1:** Description of tomato maturity detection dataset.

The Tomato Quality Grading Dataset was generated at home by capturing images of tomatoes at different stages every three days, with the main factors determining tomato quality being color, texture, and flavor. [4], Table 2 below provides information about fresh and defective classes within the Tomato Quality Grading Dataset.

**Table 2:** Description of tomato quality grading dataset.

## 3 Experimental Design, Materials and Methods

### 3.1 Camera specification

The dataset was collected using three different smartphones: Samsung Galaxy, Redmi Note-9, and Redmi Y3. Each of these smartphones has specific camera configurations. The Samsung Galaxy is equipped with a triple-lens reflex digital camera that includes a variety of lenses whereas the Redmi Note-9 has a quad-lens reflex digital camera with different lens types. Additionally, the Redmi Y3 features a dual-lens reflex digital camera. All of these cameras come with HDR functionalities, panorama, and LED flash.

### 3.2 Data augmentation

In order to satisfy the needs of machine vision-based deep learning models, which require a significant number of images, we applied data augmentation methods. Data augmentation serves the purpose of enlarging the dataset size, mitigating overfitting, and enhancing the overall performance of deep learning models [5]. This technique involves actions like rotations, zooming, and mirroring.

During the augmentation process, we carefully adjusted specific parameters, including a probability of 0.07 with maximum left and right rotation angles of 10°, and a probability of 1 with maximum left and right rotation angles of 5° for the rotation component. For zoom and random zoom, the parameters included a probability of 0.5, a minimum factor of 1.1, a maximum factor of 1.5, and a probability of 0.5 with a percentage area of 0.08.

**Fig. 2:**
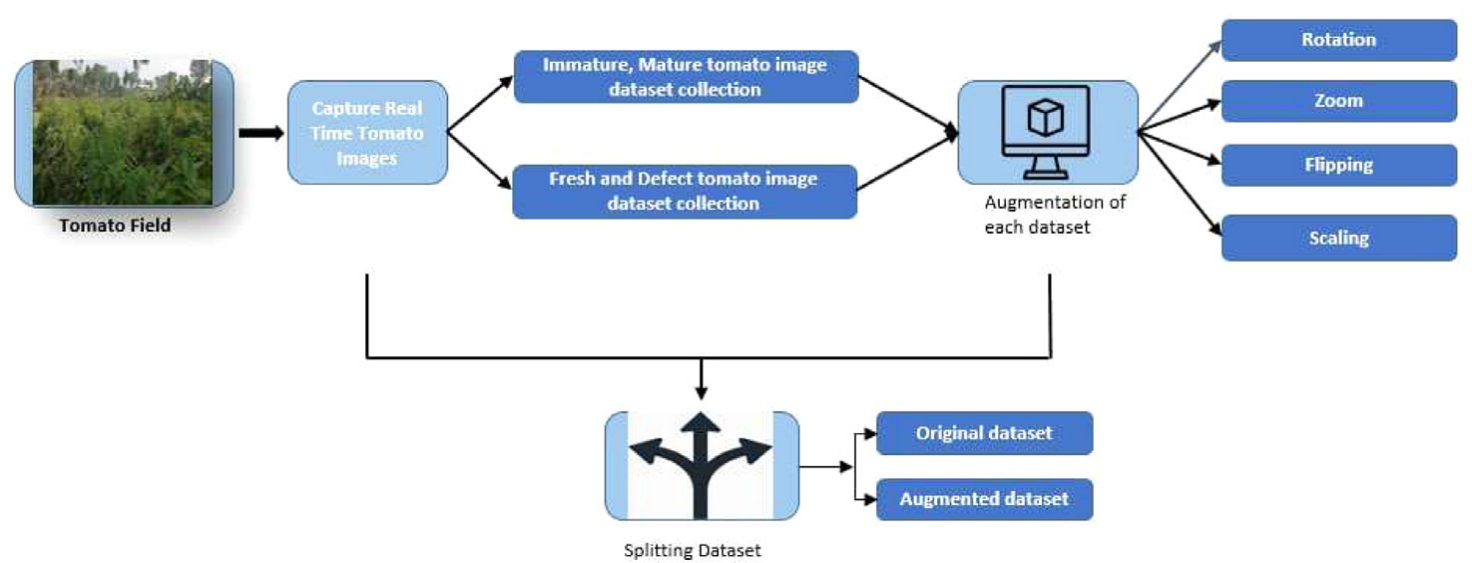
The process of tomato dataset generation.

The augmentation process was executed automatically using software, resulting in the creation of 10,000 augmented images derived from the original dataset. Fig. 2 provides a visual representation of the tomato dataset generation process. Furthermore, Tables 3 and 4 display the augmented images alongside their corresponding original sample images in each category.

**Table 3:** Augmented images of tomato maturity detection dataset.

**Table 4:** Augmented images of tomato quality grading dataset.

Detailed statistics regarding the image dataset can be found in Table 5.

**Table 5:** The statistics of the tomato dataset.

Dataset category	Class	No of image in the original dataset	No of image in the augmented dataset
Tomato maturity detection dataset	Immature	500	2000
	Mature	500	2000
Tomato quality grading dataset	Fresh	1350	3000
	Defect	636	3000

### 3.3 Deep learning model validation

We proposed a deep learning model aimed at efficiently training the dataset to achieve state-of-the-art results. The validation of a deep learning model necessitates a meticulous examination of its output on a dataset, as discussed in [6]. The deep learning model adheres to a five-stage procedure, which involves data preprocessing, data partitioning, model training, assessing performance using a validation set, and finally, testing the model on an entirely separate test set. This rigorous methodology is essential to confirm the model's trustworthiness in delivering precise outcomes and its capability to generalize to new data.

Effective data preprocessing plays a pivotal role in extracting valuable insights from the dataset. In our study, the preprocessing of images encompasses various data transformations, which include tasks like image resizing, contrast enhancement, noise reduction, augmentation, and segmentation. The specific details are outlined below:

*Noise reduction:* We employed the non-linear median filtering technique to effectively remove noise from the images. This choice was made due to the impulsive nature of the noise observed.

*Contrast enhancement:* To rectify uneven illumination and enhance image contrast, we utilized the histogram equalization technique.

*Image resizing:* Since images in the dataset may exhibit varying sizes, we deemed it necessary to resize them as per our requirements. This step was essential to ensure compatibility when training deep learning models.

*Image segmentation:* When necessary, we performed image cropping to eliminate unwanted background elements, enhancing the quality of the dataset.

*Data augmentation:* To augment our dataset, a crucial requirement for training deep learning models, we implemented data augmentation techniques. Further details of this augmentation process can be found in Section 3.2.

We partitioned the gathered data into two distinct sets: the training set and the testing set, in an 80:20 ratio. Specifically, 80% of the images were randomly selected for the training dataset, while the remaining 20% constituted the test dataset. The testing set served the purpose of evaluating the model's performance once it had been trained using the training data.

**Fig. 3:**
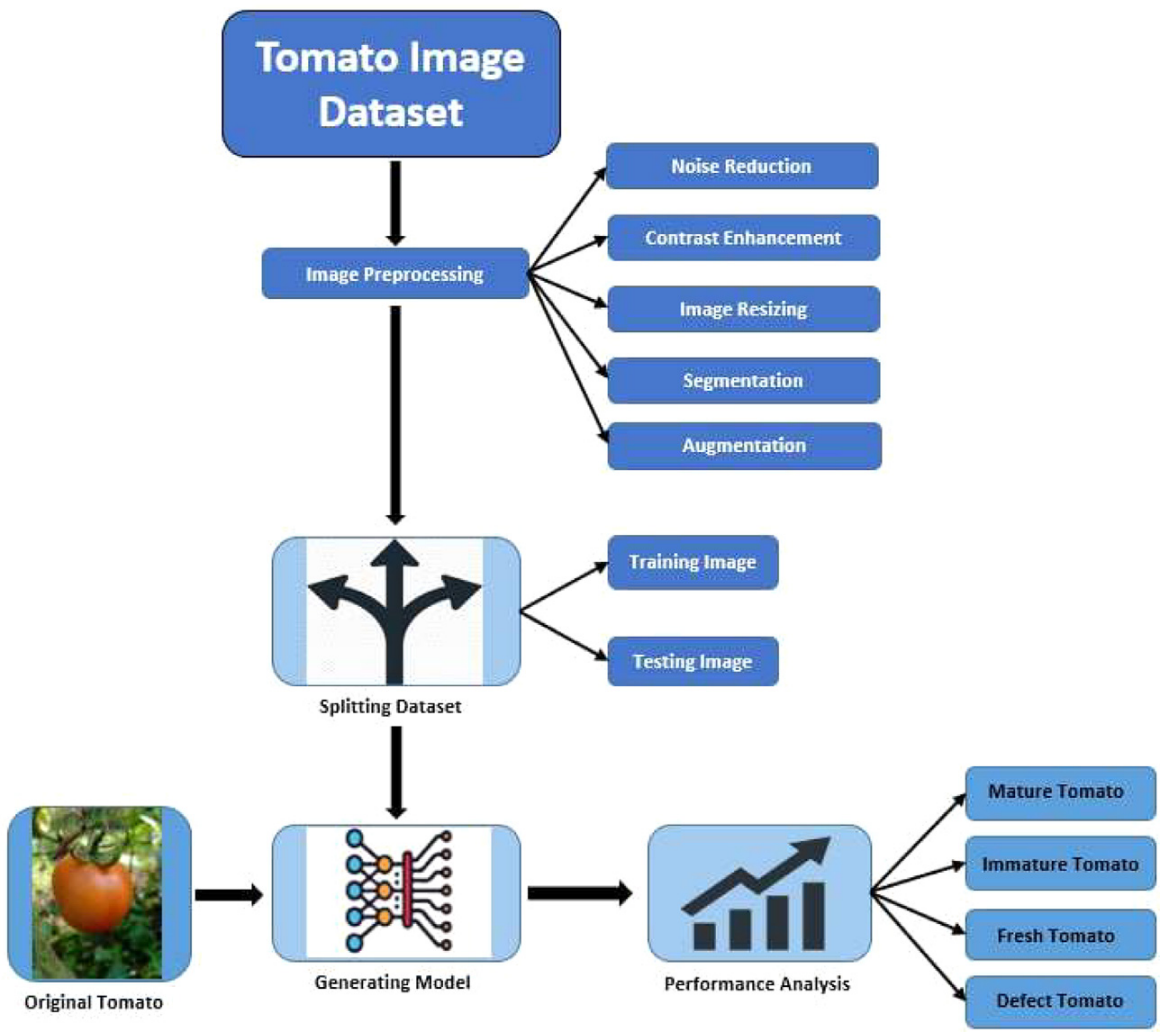
Working procedure of maturity detection and the recognition of fresh and defect tomatoes.

Fig. 3 provides an overview of the extensive validation procedures applied to our deep-learning model using the tomato image dataset. This validation encompassed tasks such as distinguishing between mature and immature tomatoes and classifying tomatoes as fresh or defective.

#### 3.3.1 Model description

In this paper, we have implemented the MobileNetV2 architecture for the purpose of detecting tomato maturity and quality. MobileNetV2 is a convolutional neural network (CNN) architecture explicitly crafted for efficient image classification purposes [7]. It builds upon the original MobileNetV2 architecture by introducing innovative architectural elements, prominently featuring inverted residuals. These residuals are composed of the following key components:

• Depth-wise convolution: This component entails the application of a separate 3×3 convolution to each input channel. This operation effectively captures spatial information within the data.
• Point-wise convolution: Following the depth-wise convolution, a 1×1 convolution is applied to amalgamate the output channels. This step serves a dual purpose by reducing dimensionality and introducing non-linearity into the network.

**Fig. 4:**
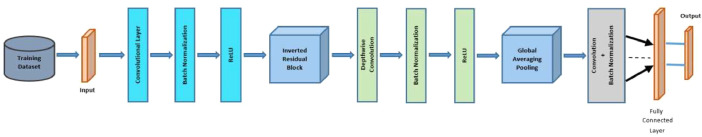
MobileNetV2 architecture.

In MobileNetV2, batch normalization is applied before activation functions (e.g., ReLU) within each convolutional or fully connected layer. MobileNetV2 typically employs global average pooling to reduce the spatial dimensions of the feature maps. This step converts the feature maps into a fixed-size vector. Fig. 4 represents MobileNetV2 architecture.

#### 3.3.2 Measurement metrics

*Confusion matrix:* A confusion matrix stands as a crucial instrument in the realm of machine learning and classification endeavors. It serves the purpose of appraising a predictive model's performance. By employing a confusion matrix, one can compute a range of performance indicators like accuracy, precision, recall, and F1 score, which aid in gauging the model's proficiency in accurately categorizing instances and pinpointing potential origins of errors, such as false positives and false negatives.

*Accuracy:* Accuracy, a crucial performance metric in classification, quantifies the fraction of accurately classified instances within the dataset. This metric is determined by dividing the count of correct predictions (comprising both true positives and true negatives) by the total number of instances. Accuracy offers a general evaluation of a model's correctness. It is imperative to also take into account precision, recall, and the F1 score in conjunction with accuracy to gain a comprehensive insight into a model's performance.

(1) $$\text{Accuracy}=\frac{\text{TP}+\text{TN}}{\text{TP}+\text{FP}+\text{TN}+\text{FN}}$$

Where

True Positive (TP) - the classifier classifies the right class of tomato as right

True Negative (TN) - the classifier classifies the wrong class of tomato as wrong

False Positive (FP) - the classifier classifies the wrong class of tomato as right

False Negative (FN) - the classifier classifies the right class of tomato as wrong

*Precision:* Precision, within the realm of classification tasks, is a performance measure that gauges the precision of positive predictions made by a model. It is calculated as the proportion of true positive predictions relative to the total number of positive predictions (which includes both true positives and false positives). In essence, precision evaluates the model's capacity to accurately pinpoint relevant instances among its positive forecasts. A higher precision signifies a reduced occurrence of false positives and, consequently, a decreased likelihood of incorrectly classifying negative instances as positive.

(2) $$\text{Precision}=\frac{\text{True}\;\text{Positives}}{\text{True}\;\text{positives}+\text{False}\;\text{Positives}}$$

*Recall:* Recall, also known as true positive rate, quantifies a model's aptitude in accurately recognizing all pertinent instances within a dataset. It is expressed as the fraction of true positive predictions relative to the total number of actual positive instances (comprising both true positives and false negatives). Recall becomes especially valuable when the expense associated with overlooking positive instances (false negatives) is substantial. A heightened recall signifies an increased capability to capture the majority of positive cases.

(3) $$\text{Recall}=\frac{\text{True}\;\text{Positives}}{\text{True}\;\text{positives}+\text{False}\;\text{Negatives}}$$

*F1 score:* The F1 score serves as a metric that melds together both precision and recall to deliver a well-rounded evaluation of a model's performance. Its computation involves taking the harmonic mean of precision and recall, affording equal importance to both metrics. The F1 score proves particularly valuable when there exists an uneven distribution between the positive and negative classes in the dataset. It offers a single numerical representation that takes into account both false positives and false negatives, establishing it as a dependable gauge of the overall classification performance.

(4) $$F-1\;\text{Score}=\frac{2*\left(\text{Precision}*\text{Recall}\right)}{\text{Precision}+\text{Recall}}$$

**Fig. 5:**
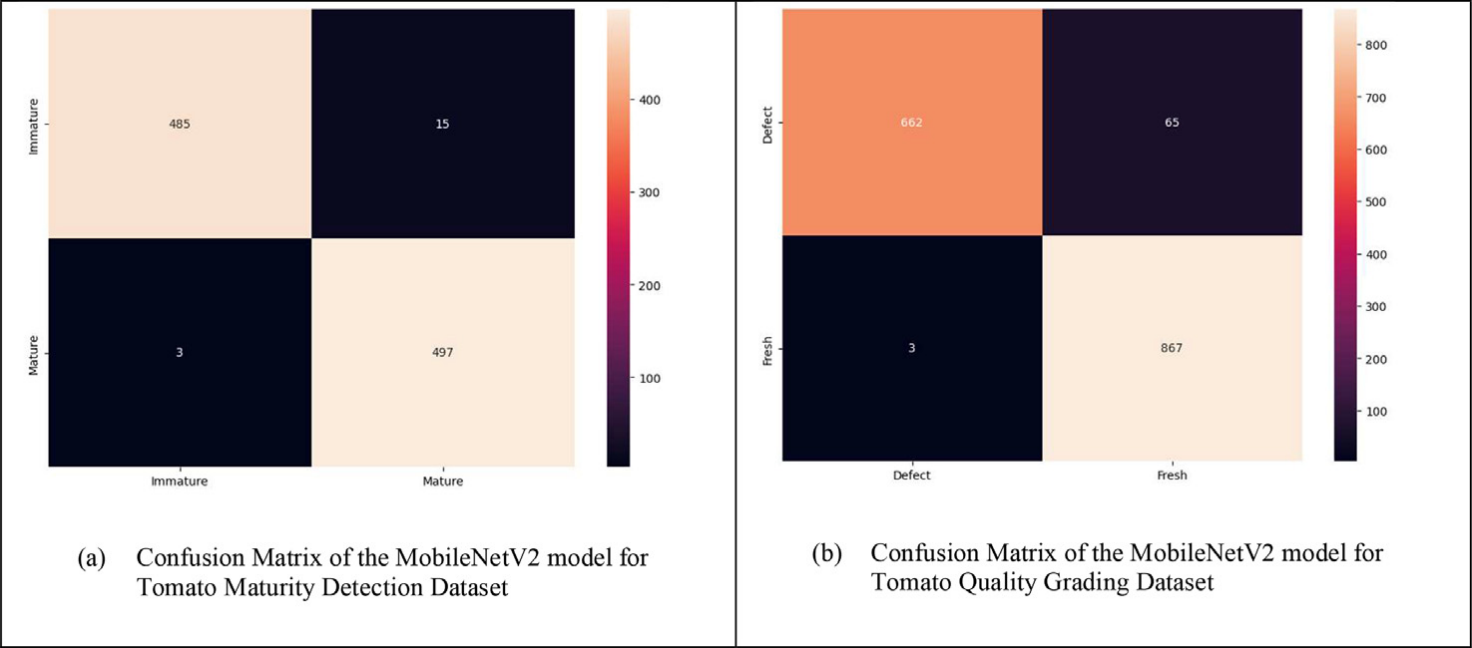
Confusion matrices of the MobileNetV2 model

The confusion matrices of the MobileNetV2 model is shown in Fig. 5.

Table 6 represents the performance metrics for the MobileNetV2 model.

**Table 6:** Performance metrics for MobileNetV2 model.

Classes	Model	Precision	Recall	F-1 score	Accuracy
Immature	MobileNetV2	0.99	0.97	0.98	98%
Mature		0.97	0.99	0.98	
Fresh	MobileNetV2	0.93	1	0.96	96%
Defect		1	0.91	0.95	

In the future, we will extensively examine more state-of-the-art deep learning models using this dataset to determine the best technique for practical applications.

## Limitations

This system is built to focus only on Tomato data.

## Ethics Statement

None of the authors of this article have conducted any research using humans or animals as subjects. The datasets consulted for this article are accessible to everyone but following the correct citation guidelines is essential.

## CRediT authorship contribution statement

**Tania Khatun:** Conceptualization, Data curation, Methodology, Visualization, Validation, Writing – original draft, Writing – review & editing. **Abdur Razzak:** Methodology, Data curation. **Md. Shofiul Islam:** Methodology, Data curation. **Mohammad Shorif Uddin:** Supervision, Writing – review & editing.

## Data Availability

Tomato Maturity Detection and Quality Grading Dataset (Original data) (Mendeley Data).
